# Intuitive eating was associated with anxiety, depression, pregnancy weight and blood glucose in women with gestational diabetes mellitus: a prospective longitudinal study

**DOI:** 10.3389/fnut.2024.1409025

**Published:** 2024-07-25

**Authors:** Xiao xia Gao, Qing xiang Zheng, Xiao qian Chen, Xiu min Jiang, Yan ping Liao, Yu qing Pan, Jing jing Zou, Gaoqian Liu

**Affiliations:** ^1^Fujian Maternity and Child Health Hospital College of Clinical Medicine for Obstetrics and Gynecology and Pediatrics, Fujian Medical University, Fuzhou, China; ^2^Guangzhou Women and Children's Medical Center, Guangzhou Medical University, Guangzhou, China; ^3^Mindong Hospital Affiliated to Fujian Medical University, Ningde, China; ^4^Lishi District Changzhi Road Primary School, Lüliang, China

**Keywords:** intuitive eating, anxiety, depression, GDM, weight, blood glucose

## Abstract

**Background:**

Outside of pregnancy, intuitive eating (IE) is associated with lower body weight, blood glucose, and higher positive mood. However, little was known about the relationship between IE and anxiety-depression in the GDM population. Thus, this study aimed to investigate the association of IE with anxiety and depression, pregnancy weight and pregnancy blood glucose in the first and second GDM visit.

**Methods:**

Data from 310 pregnant women with GDM from the Fujian Maternal and Child Health Hospital Trial (Approval Number: 2020Y9133) were analyzed. IE was assessed using the Intuitive Eating Scale-2 subscales of Eating for Physiological Reasons rather than Emotional Reasons (EPR), Relying on Hunger and Satiety Cues (RHSC) and Body-Food Choice Consistency (B-FCC). Observations included weight, body mass index (BMI), fasting plasma glucose (FPG) and 2-h postprandial blood glucose; the Hospital Anxiety and Depression Scale (HADS) was used to assess the level of anxiety and depression in pregnant women with GDM. Linear regression analysis was used to assess the correlation between IE and anxiety, depression, pregnancy blood glucose and weight.

**Results:**

The cross-sectional analysis showed that the EPR eating behavior was negatively correlated with anxiety and depression, and the B-FCC eating behavior was negatively correlated with depression at both the first and second GDM visit; in addition, the B-FCC eating behavior was associated with lower BMI in the third trimester (all *p* < 0.05). In longitudinal analyses, the EPR eating behavior in the first visit for GDM predicted lower levels of anxiety and depression in the second GDM visit, whereas the RHSC eating behavior in the first visit for GDM was associated with lower FPG in the second GDM visit (all *p* < 0.01).

**Conclusion:**

These results suggest that practicing intuitive eating may be beneficial and that higher intuitive eating adherence can lead to lower levels of anxiety and depression and more ideal gestational weight and blood glucose values.

## 1 Background

Gestational diabetes mellitus (GDM), defined as glucose intolerance with onset or first recognition in pregnancy, was a common complication of pregnancy (1). The global prevalence of GDM was about 14% (2), it can be as high as 21% in China (3), and the prevalence was increasing (4, 5). GDM was associated with adverse maternal and neonatal outcomes, and the risk of these outcomes increases with increasing fasting plasma glucose levels (6). In the short term, women with GDM had an increased risk of preeclampsia, gestational hypertension and cesarean section, and had a higher incidence of macrosomia, shoulder dystocia, neonatal hypoglycemia, respiratory distress syndrome and neonatal intensive care admission (7–9). In the long term, there was an increased risk for metabolic dysfunction for both mother and infant including diabetes, obesity and metabolic syndrome (10, 11). In addition, women with GDM had higher levels of anxiety and depression (12), and the diagnosis of GDM increased their susceptibility to depression or anxiety (13). Their risk of depression was 2–4 times higher than that of normal pregnant women (14).

The mainstay of treatment for GDM was dietary intervention, and better pregnancy outcomes were linked to adhering to dietary recommendations for diabetes (15). However, it was unable to determine which kind of dietary advice was most appropriate for pregnant women with GDM since there were 50 various types of recommendations which were evaluated by a systematical review study (16). Previous studies has demonstrated that women with GDM experienced many difficulties such as conflicts between personal food preferences and dietary recommendations, the need for an immediate dietary change, low suitability and compliance with recommendations, and a lack of favorable factors to support implementation (17–20). Overly strict dietary control and detachment from cultural realities during the period of following dietary recommendations caused them to feel great frustration and psychological burden, further reducing adherence to dietary therapy (21, 22). Therefore, it was vital to explore suitable and conducive to the physical and mental health of GDM women.

**Figure 1:**
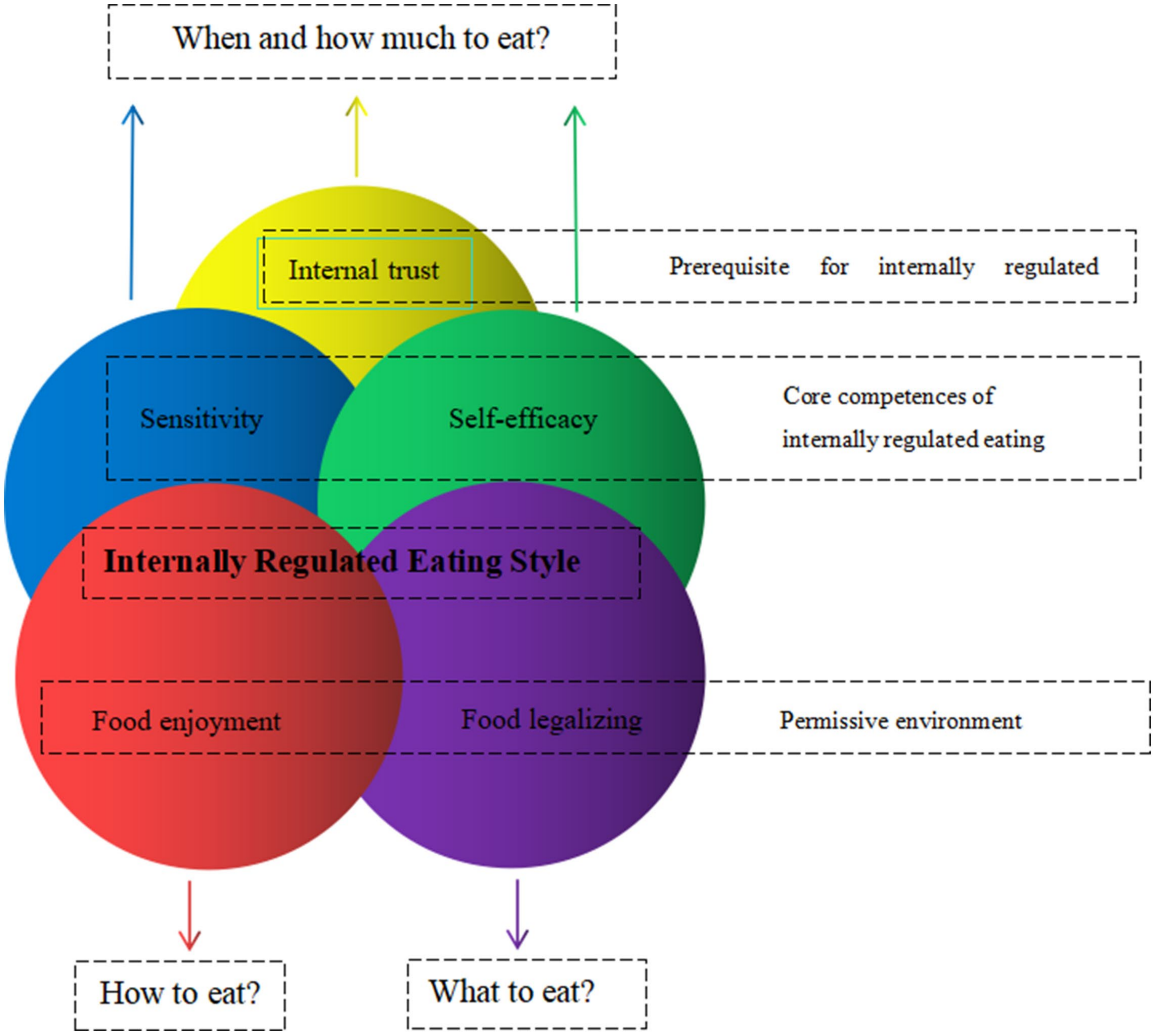
This theoretical framework of internal dietary regulation.

Intuitive eating was defined as a “dynamic process that integrates the coordination of mind, body and food.” It was essential to the regulation of food intake based on hunger and satiety signals, following the body wisdom that the body was able to sense how much and what kind of food to eat in order to maintain nutritional health and appropriate weight. It was an internal adaptive eating behavior that was beneficial to physical and mental health, and could be adhered to for a long time (23). This theoretical framework of internal dietary regulation is characterized by five main individual differences: Internal trust, Sensitivity, Self-efficacy, Food enjoyment, and Food legalizing (see Figure 1) (24). Previous studies had shown that intuitive eating was beneficial to reduce weight loss (25), maintain a lower Body mass index (BMI) (26), control blood glucose (27), reduce triglyceride levels and the risk of cardiovascular disease (28); and positively correlated with psychological constructs such as positive body image, self-esteem and well-being (29).

Much of the existing evidence on intuitive eating in the maternal population was almost based on cross-sectional studies. High intuitive eating was positively correlated with improved diet quality, positive attitudes toward gestational weight gain, and postpartum body image satisfaction. It was also associated with lower gestational weight gain, restricted eating, postpartum weight loss, and postpartum depression (30–34). The few longitudinal studies that had followed women with GDM from their first GDM visit to 6–8 weeks postpartum or 1 year postpartum had found that intuitive eating was associated with lower fasting plasma glucose (FPG) and Hemoglobin A1C (HbA1C) in the first GDM visit and the postpartum (35, 36). However, it was still unknown how intuitive eating would affect GDM women’ physical and mental health (such as anxiety, depression, and pregnancy weight) during pregnancy. Therefore, the aim of this study was to investigate the cross-sectional and longitudinal associations between intuitive eating and blood glucose, weight, anxiety, and depression in women with GDM during pregnancy.

## 2 Methods

### 2.1 Study design and patient population

**Figure 2:**
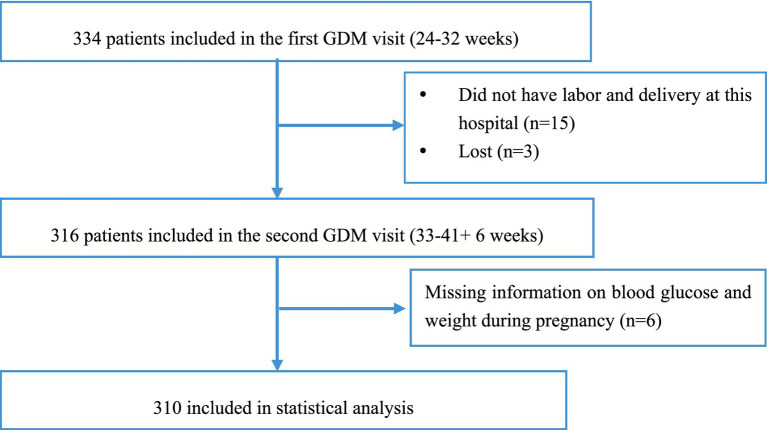
Flow chart of study participants inclusion.

This study was based on data from a cohort study of pregnant women with GDM conducted at Fujian Maternity and Child Health Hospital (Approval Number: 2020Y9133). Pregnant women diagnosed with GDM according to the International Association of Diabetes and Pregnancy Study Groups (IADPSG) and American Diabetes Association (ADA) guidelines (37, 38), who were invited to participate in this study who were ≥18 years old, completed the intuitive eating questionnaire at the time of their first GDM visit (i.e., the first visit after GDM diagnosis and diabetic dietary guidance was not initiated), and were not undergoing insulin and other medication. From the cohort population of 334 participants followed up at our hospital, we excluded 24 participants. Overall, 310 GDM women were included in the final analysis (see Figure 2).

### 2.2 Data collection

This research mainly divided into two periods to collect questionnaire. (1) The first GDM visit during the second trimester: the researchers and the trained investigators explained the purpose and significance of the study and the method of filling out the questionnaire to the study subjects. By looking at the clinic electronic medical records, double check and record height, the weight and gestational age, OGTT results and blood glucose, etc.; Add the object of study WeChat, mobile phone number and other contact information, to prepare for the third trimester follow-up survey. (2) The second GDM visit during the third trimester: for the first time by the same investigators into the object of study in the third trimester paper face to face questionnaire survey and back on the spot; track to pregnancy outcomes, by looking at the hospital medical records, double check and record in hospital after the delivery for the first time measuring body weight, FPG, 2-h postprandial glucose after meal and the correlative data.

#### 2.2.1 General information questionnaire

The general information questionnaire included age, weight, education level, occupation, family *per capita* monthly income, family history of obesity and disease, sleep and exercise, etc.

#### 2.2.2 Assessment of intuitive eating

We assessed intuitive eating with the Intuitive Eating Scale-2 (IES-2) (23). It is a 23-item questionnaire assessing four dimensions of intuitive eating as well as providing a global score. Four subscales include: (1) Unconditional Permission to Eat (UPE, 6 items), that assesses whether an individual purposefully tries to ignore hunger and satiety signals; (2) Eating for Physical Rather than Emotional Reasons (EPR, 8 items), that assesses how much eating is affected by emotional responses; (3) Reliance on Hunger and Satiety Cues (RHSC, 6 items), that evaluates the extent to which individuals are aware and able to trust internal signals rather than relying on external rules/cues, and (4) Body-Food Choice Congruence (BFC-C, 3 items), that assesses the degree of consistency demonstrated between physical needs and food choices while satisfying taste buds. Items are rated on a 5-point Likert scale ranging from 1 (strongly disagree) to 5 (strongly agree). Mean scores are calculated for the subscales and the global score, with higher scores indicating greater intuitive eating. The Cronbach’s α values for the IES-2 and its four subscales ranged from 0.81 to 0.93.

For the purpose of our study, we removed the UPE subscale (4 items) from the IES-2. Although we measured intuitive eating before the diabetic dietary guidance we did not include the UPE subscale in this study, because the diagnosis of GDM itself and subsequent dietary counseling could significantly influence responses to the UPE subscale questions, such as “I try to avoid certain foods high in fat, carbohydrates, or calories.” However, we measured intuitive eating before the dietician visit to ensure that, diabetic dietary guidance did not influence study outcomes.

#### 2.2.3 Blood glucose and weight during pregnancy

We extracted data on FPG, HbA1c and 2-h postprandial glucose from mothers’ medical records in the first and second GDM visits, which were measured at the time of completing the intuitive eating questionnaire.

Pre-pregnancy weight was extracted from participants’ medical charts or, if missing, was self-reported. We measured height and weight in the first GDM visit during pregnancy, as well as weight in the third trimester to the nearest 0.1 cm and 0.1 kg with electronic scales. BMI was expressed as a ratio of weight in kilograms to the square of height in meters (kg/m2).

#### 2.2.4 Assessment of anxiety and depression

Anxiety and depression were assessed utilizing Hospital Anxiety and Depression Scale (HADS) (39). It comprises 14 questions: seven associated with the anxiety evaluation (HADS-A) and seven associated with the depression evaluation (HADS-D). Each item was rated on a 4-point scale ranging from 0 to 3. For both HADS-A and HADS-D, we divided the respondents into subgroups by using a cut-off of ≥8 points for depression or anxiety to define pathologic and non-pathologic values, according to the recommendations in the literature. Since the total score for each subscale is 21, a total score greater than 8 for each subscale indicates that the person may have symptoms of anxiety or depression. The Cronbach’s α values for the HADS, HADS-A subscales and HADS-D subscales were 0.879, 0.806 and 0.806, respectively (40).

### 2.3 Statistical analyses

All analyses were conducted using the SPSS software version 20 (IBM Corp., 2012). All descriptive variables were presented as either means (±standard deviation) or in percentages (%) where appropriate. Both predictor (EPR, RHSC, and BFC-C subscales of the IES-2 questionnaire) and outcome (BMI, weight, FPG, 2-h postprandial glucose, HbA1c, HADS-A, and HADS-D at the different time points) variables were normally distributed. So we used paired *t*-tests or Wilcoxon test to determine changes in EPR, RHSC, BCFF, HADS, and maternal glucose between the first and second GDM visit. We used linear regression analyses to determine the associations between EPR, RHSC, and BCFF with HADS, blood glucose and pregnancy weight, namely the cross-sectional association between EPR, RHSC, and BCFF with HADS, pregnancy blood glucose and pregnancy weight in the first and second GDM visit, and longitudinal associations between EPR, RHSC, and BCFF in the first GDM visit with HADS, pregnancy weight and blood glucose the second GDM visit.

## 3 Results

### 3.1 Baseline characteristics of participants

Table 1 shows the socio-demographic characteristics of the participants. The mean age of participants was (31.85 ± 3.95) years and the mean gestational age in first GDM visit was (26.37 ± 1.94) weeks. The mean pre-pregnancy BMI was (22.63 ± 3.40) kg/m2 and BMI was (25.40 ± 3.47) kg/m^2^ in the first GDM visit. Half (50.3%) of the participants were university graduates, and 73.5% were of employees. Few women had a history of previous GDM (9.4%) and a third (32.3%) of the participants had a family history of diabetes.

**Table 1:** Socio-demographic characteristics of study participants (*n* = 310).

Variable	Mean ± SD/*n* (%)
Age (year)	31.85 ± 3.95
Gestational age in the first GDM visit (weeks)	26.37 ± 1.94
Weight before pregnancy (kg)	57.80 ± 9.68
BMI before pregnancy (kg/m^2^)	22.63 ± 3.40
Weight in first GDM visit (kg)	64.81 ± 9.78
BMI in first GDM visit (kg/m^2^)	25.40 ± 3.47
Weight in the third trimester (kg)	67.78 ± 10.00
BMI in the third trimester (kg/m^2^)	26.58 ± 3.64
**Educational level, *n* (%)**	
Compulsory school achieved	30 (9.7%)
High school	39 (12.9%)
General and vocational education	85 (27.4%)
University	156 (50.3%)
**Employment status, *n* (%)**	
Employed	228 (73.5%)
Unemployed/housewife	82 (26.5%)
**Preconception exercise, *n* (%)**	
None	74 (23.9%)
1–2 days/week	147 (47.4%)
3–5 days/week	89 (28.7%)
6–7 days/week	0
**Family history of diabetes, *n* (%)**	
Yes	100 (32.3%)
No	210 (67.7%)
**History of GDM, *n* (%)**	
Yes	29 (9.4%)
No	281 (90.6%)
**Gravida, *n* (%)**	
1	131 (42.3%)
2	99 (31.9%)
≥3	80 (25.8%)
**Parity, *n* (%)**	
0	187 (60.3%)
1	100 (32.3%)
2	22 (7.1%)
≥3	1 (0.3%)

GDM denotes gestational diabetes mellitus; BMI denotes body mass index.

**Figure 3:**
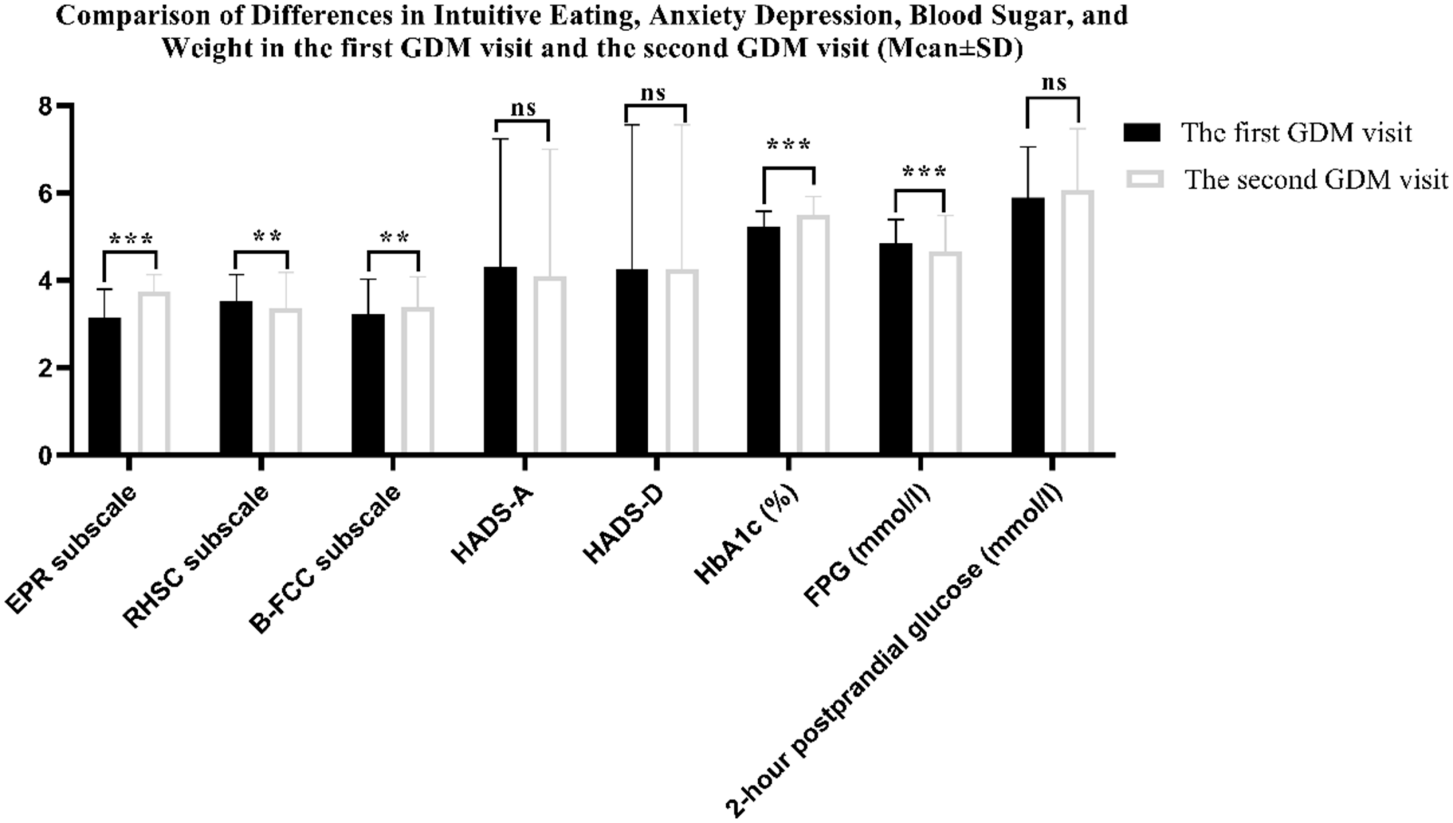
Comparison of Differences in Intuitive Eating, Anxiety Depression, Blood Sugar, and Weight in the first GDM visit and the second GDM visit (***denotes *p* < 0.001; **denotes *p* < 0.01; ns denotes *p* > 0.05).

Figure 3 shows the change in intuitive eating, pregnancy weight and blood glucose variables between the first GDM visit and the second GDM visit. There were differences in the three subscales of the intuitive eating HbA1c and FPG between the two periods (all *p* < 0.01). However, anxiety, depression and 2-h postprandial glucose were not statistically significant (*p* > 0.05). The incidences of anxiety and depression were 11.3 and 10.3% in the first GDM visit, 13.8 and 18.1% in the second GDM visit, respectively. The incidence of depression was increased in the second GDM visit compared with the first GDM visit (*p* = 0.001) (see Table 2).

**Table 2:** Differences in anxiety and depression at baseline and end of study (*n* = 310).

Variable	Group	The presence of anxiety and depression (*n*, %)		*Z*	*p*-value
		No	Yes		
HADS-A	The first GDM visit	275 (88.7)	35 (11.3)	−1.180	0.238
	The second GDM visit	267 (86.2)	43 (13.8)		
HADS-D	The first GDM visit	278 (89.7)	32 (10.3)	−3.394	0.001
	The second GDM visit	245 (81.9)	65 (18.1)		

HADS-A denotes the anxiety subscale of the HADS; HADS-D denotes the depression subscale of the HADS.

Table 3 shows the cross-sectional analyses in the first GDM visit, none of the subscales of intuitive eating was associated with pregnancy weight and pregnancy blood sugar; however, the EPR eating behavior were associated with lower anxiety (*β =* −1.107, 95% CI: −1.587, −0.627) and depression (*β =* −1.084, 95% CI: −1.563, −0.604), the B-FCC eating behavior was associated with lower depression (*β =* −0.560, 95% CI: −0.968, −0.151) (all *p* < 0.05). In the cross-sectional analyses in the second GDM visit, the RHSC eating behavior was not associated with any of the observed indicators; while, the EPR eating behavior were associated with lower anxiety (*β =* −0.949, 95% CI: −1.563, −0.604) and depression (*β =* −1.284, 95% CI: −1.707, −0.860); the B-FCC eating behavior was associated with lower depression (*β =* −0.859, 95% CI: −1.363, −0.354) and BMI (*β =* −0.616, 95% CI: −1.211, −0.022) (all *p* < 0.05).

**Table 3:** Cross-sectional associations between the three subscales of IES-2 and weight, BMI and glucose in the first GDM visit and the second GDM visit (*n* = 310).

Variable	Effect estimate		
In the first GDM visit during the second trimester	Standardized beta coefficient	*β* (95% CI)	*p-*value
**EPR**			
HADS-A in the first GDM visit	0.244	−1.107 (−1.587, −0.627)	<0.001
HADS-D in the first GDM visit	0.244	−1.084 (−1.563, −0.604)	<0.001
Weight before pregnancy (kg)	0.855	−0.381 (−2.064, 1.301)	0.656
BMI before pregnancy (kg/m^2^)	0.330	−0.103 (−0.694, 0.487)	0.731
Weight in the first GDM visit (kg)	0.863	−0.744 (−2.442, 0.954)	0.389
BMI in the first GDM visit (kg/m^2^)	0.306	−0.237 (−0.840, 0.366)	0.440
HbA1c in the first GDM visit (%)	0.024	−0.009 (−0.056, 0.039)	0.722
FPG in the first GDM visit (mmol/l)	0.038	−0.027 (−0.103, 0.049)	0.487
2-h postprandial glucose in the first GDM visit (mmol/l)	0.082	0.073 (−0.087, 0.233)	0.371
**RHSC**			
HADS-A in the first GDM visit	0.267	0.348 (−0.178, 0.874)	0.194
HADS-D in the first GDM visit	0.267	0.382 (−0.144, 0.908)	0.154
Weight before pregnancy (kg)	0.937	−0.275 (−2.118, 1.569)	0.770
BMI before pregnancy (kg/m^2^)	0.329	−0.004 (−0.651, 0.644)	0.991
Weight in the first GDM visit (kg)	0.946	−0.149 (−2.010, 1.712)	0.875
BMI in the first GDM visit (kg/m^2^)	0.336	0.038 (−0.623, 0.698)	0.910
HbA1c in the first GDM visit (%)	0.025	−0.002 (−0.051, 0.047)	0.941
FPG in the first GDM visit (mmol/l)	0.040	−0.046 (−0.124, 0.032)	0.248
2-h postprandial glucose in the first GDM visit (mmol/l)	0.084	0.023 (−0.143, 0.188)	0.789
**B-FCC**			
HADS-A in the first GDM visit	0.208	−0.109 (−0.518, 0.300)	0.600
HADS-D in the first GDM visit	0.208	−0.560 (−0.968, −0.151)	0.007
Weight before pregnancy (kg)	0.728	−0.333 (−1.766, 1.100)	0.648
BMI before pregnancy (kg/m^2^)	0.256	−0.043 (−0.547, 0.460)	0.866
Weight in the first GDM visit (kg)	0.735	−0.421 (−1.868, 1.025)	0.567
BMI in the first GDM visit (kg/m^2^)	0.261	−0.061 (−0.574, 0.574)	0.815
HbA1c in the first GDM visit (%)	0.029	0.006 (−0.051, 0.062)	0.846
FPG in the first GDM visit (mmol/l)	0.046	0.005 (−0.085, 0.096)	0.906
2-h postprandial glucose in the first GDM visit (mmol/l)	0.097	0.025 (−0.166, 0.216)	0.796
**In the second GDM visit during the third trimester**	**Standardized beta coefficient**	***Β* (95% CI)**	***p*-value**
**EPR**			
HADS-A in the second GDM visit	0.197	−0.949 (−1.336, −0.562)	<0.001
HADS-D in the second GDM visit	0.215	−1.284 (−1.707, −0.860)	<0.001
Weight in the second GDM visit (kg)	0.697	0.432 (−0.939, 0.804)	0.536
BMI in the second GDM visit (kg/m^2^)	0.253	0.312 (−0.186, 0.811)	0.219
HbA1c in the second GDM visit (%)	0.029	0.006 (−0.05, 0.063)	0.825
FPG in the second GDM visit (mmol/l)	0.057	−0.029 (−0.141, 0.083)	0.609
2-h postprandial glucose in the second GDM visit (mmol/l)	0.117	−0.042 (−0.235, 0.151)	0.668
**RHSC**			
HADS-A in the second GDM visit	0.203	−0.007 (−0.407, 0.393)	0.974
HADS-D in the second GDM visit	0.223	−0.039 (−0.477, 0.399)	0.862
Weight in the second GDM visit (kg)	0.720	1.309 (−0.108, 2.727)	0.070
BMI in the second GDM visit (kg/m^2^)	0.262	0.418 (−0.098, 0.933)	0.112
HbA1c in the second GDM visit (%)	0.030	−0.004 (−0.062, 0.054)	0.894
FPG in the second GDM visit (mmol/l)	0.059	−0.059 (−0.175, 0.056)	0.313
2-h postprandial glucose in the second GDM visit (mmol/l)	0.101	−0.145 (−0.344, 0.054)	0.153
**B-FCC**			
HADS-A in the second GDM visit	0.234	−0.399 (−0.861, 0.062)	0.090
HADS-D in the second GDM visit	0.257	−0.859 (−1.363, −0.354)	0.001
Weight in the second GDM visit (kg)	0.831	−1.562 (−3.196, 0.073)	0.061
BMI in the second GDM visit (kg/m^2^)	0.302	−0.616 (−1.211,-0.022)	0.042
HbA1c in the second GDM visit (%)	0.034	0.034 (−0.033, 0.101)	0.323
FPG in the second GDM visit (mmol/l)	0.068	−0.110 (−0.243, 0.023)	0.105
2-h postprandial glucose in the second GDM visit (mmol/l)	0.117	−0.098 (−0.327, 0.132)	0.404

EPR denotes Eating for Emotional Rather than Physical reasons of the French Intuitive eating Scale-2 (IES-2); RHSC denotes Reliance on Hunger and Satiety subscale of the IES-2; B-FCC denotes reliance on hunger and satiety cues of the IES-2; HADS-A denotes the anxiety subscale of the HADS; HADS-D denotes the depression subscale of the HADS; HbA1c means glycated hemoglobin, fasting plasma glucose means FPG.

In the longitudinal analyses (Table 4), the EPR eating behavior in the first GDM visit was associated with lower anxiety (*β =* −0.991, 95% CI: −1.486, −0.496) and depression (β = −1.081, 95% CI: −1.639, −0.523) in the second GDM visit; the RHSC eating behavior in the first GDM visit was associated with FPG in the second GDM visit (all *p* < 0.001).

**Table 4:** Longitudinal associations between three subscales of IES-2 and weight, BMI and glucose in the first GDM visit and the third trimester.

Variable	Effect estimate		
EPR Subscale in the first GDM visit	Standardized beta coefficient	*Β* (95% CI)	*p-*value
HADS-A in the third trimester	0.252	−0.991 (−1.486, −0.496)	<0.001
HADS-D in the third trimester	0.284	−1.081 (−1.639, −0.523)	<0.001
Weight in the third trimester (kg)	0.883	−0.091 (−1.828, 1.646)	0.918
BMI in the third trimester (kg/m^2^)	0.322	0.008 (−0.624, 0.641)	0.979
HbA1c in the third trimester (%)	0.036	−0.015 (−0.086, 0.056)	0.677
FPG in the third trimester (mmol/l)	0.070	0.042 (−0.097, 0.18)	0.555
2-h postprandial glucose in the third trimester (mmol/l)	0.123	0.030 (−0.213, 0.272)	0.810
**RHSC Subscale in the first GDM visit**			
HADS-A in the third trimester	0.276	0.035 (−0.507, 0.578)	0.898
HADS-D in the third trimester	0.311	−0.029 (−0.641, 0.582)	0.925
Weight in the third trimester (kg)	0.967	−0.132 (−2.035, 1.771)	0.892
BMI in the third trimester (kg/m^2^)	0.352	0.020 (−0.673,0.714)	0.955
HbA1c in the third trimester (%)	0.039	−0.033 (−0.110, 0.045)	0.406
FPG in the third trimester (mmol/l)	0.077	−0.281 (−0.432, −0.129)	<0.001
2-h postprandial glucose in the third trimester (mmol/l)	0.135	0.075 (−0.191, 0.341)	0.579
**B-FCC Subscale in the first GDM visit**			
HADS-A in the third trimester	0.214	0.121 (−0.300, 0.543)	0.572
HADS-D in the third trimester	0.242	−0.208 (−0.683, 0.267)	0.390
Weight in the third trimester (kg)	0.752	−0.681 (−2.161, 0.798)	0.366
BMI in the third trimester (kg/m^2^)	0.274	−0.146 (−0.685, 0.393)	0.594
HbA1c in the third trimester (%)	0.031	0.032 (−0.028, 0.092)	0.301
FPG in the third trimester (mmol/l)	0.060	0.014 (−0.103,0.132)	0.809
2-h postprandial glucose in the third trimester (mmol/l)	0.105	−0.147 (−0.354, 0.060)	0.163

EPR denotes Eating for Emotional Rather than Physical reasons of the French Intuitive eating Scale-2 (IES-2); RHSC denotes Reliance on Hunger and Satiety subscale of the IES-2; B-FCC denotes reliance on hunger and satiety cues of the IES-2; HADS-A denotes the anxiety subscale of the HADS; HADS-D denotes the depression subscale of the HADS; HbA1c means glycated hemoglobin; fasting plasma glucose means FPG.

## 4 Discussion

This study investigated the association between the three IES-2 subscales and anxiety, depression, pregnancy weight and blood glucose in pregnant women with GDM during the first and second GDM visit. To our knowledge, this has not been previously studied in Chinese women with GDM. In this prospective cohort of women followed in a clinical setting, we found cross-sectional and longitudinal associations between the three subscales of the IES-2 (eating for physical rather than emotional reasons (EPR), reliance on hunger and satiety cues (RHSC), and body-food-choice congruence (B-FCC) subscales) with lower anxiety, depression, late-pregnancy weight, and late-pregnancy FPG in women after GDM. Specifically, the cross-sectional analysis showed that the EPR eating behavior was negatively correlated with anxiety and depression, and the B-FCC eating behavior was negatively correlated with depression in both the first GDM visit and the second GDM visit. Additionally, the B-FCC eating behavior was associated with lower BMI in the third trimester. In longitudinal analyses, the EPR eating behavior in the first GDM visit predicted lower anxiety and depression in the second GDM visit, whereas the RHSC eating behavior at the first GDM visit was associated with lower FPG in the second GDM visit.

### 4.1 Compared to previous studies

Studies have found that intuitive eating was negatively associated with depression and overweight/obesity in non-pregnant population (41), which helps to maintain weight loss and improve psychological distress such as anxiety and depression (26, 42). Compared with non-intuitive eaters, intuitive eaters had lower prevalence of high weight status and lower engagement in dieting, unhealthy weight control behaviors, and binge eating at 5-year follow-up (43). There was a more significant inverse association between intuitive eating scores and BMI in women compared to men (44). In addition, intuitive eating was also a promising non-restrictive treatment option for patients with diabetes (45). The most intuitive diet was associated with an 89% reduction in odds of inadequate glycemic control, and higher scores on the B-FCC subscale reduced participants’ chances of having this deficit, reduced by nearly 66%, regardless of their BMI (46). Higher score values on the total IES and EPR subscales were associated with lower HbA1c: HbA1c was 22% lower/whole unit increase in the total IES mean score, and HbA1c was 11% lower/whole unit increase in the mean eating score for physical rather than emotional reasons (27).

In a population of pregnant women, Daundasekara et al. (47) used a revised 15-item scale of preconception adaptive eating behaviors to assess preconception adaptive eating behaviors in pregnant women found that the EPR and the Relying on Hunger/Satiety Cues subscale were negatively correlated with perinatal depressive status and preconception BMI; higher intuitive eating habits predicted greater reductions in postpartum BMI, and increases in BMI during pregnancy. The greater the magnitude, the faster the rate of postpartum BMI decline; intuitive eating can help encourage postpartum weight loss without the weighing, measuring, recording, and evaluating dietary intake required for traditional weight loss programs, thus making it less difficult for new mothers to regain their postpartum weight (31). The EPR and RHSC subscales of the IES-2 in the first GDM visit were associated with lower pre-pregnancy weight and BMI; the EPR subscale was associated with HbA1c and FPG in the first GDM visit; and in longitudinal analyses, both subscales of the IES-2 in the first GDM visit were associated with lower end-of-pregnancy weight, BMI and FPG at 6–8 weeks postpartum (35). In the high-risk subgroup of GDM with postpartum overweight/obesity or prediabetes, intuitive eating during and after pregnancy was associated with lower BMI, weight retention, FPG, and HbA1c at 1 year postpartum (48). The results of the present study differed finding that the RHSC subscale was not associated with anxiety and depression, the EPR and RHSC eating behavior were not associated with gestational weight, and the EPR and B-FCC eating behavior were not associated with gestational blood glucose. The possible explanation is that pregnant women with GDM need to follow a diabetic diet plan for glycaemic control and are unable to perceive hunger and satiety signals. The mean weight and mean blood glucose in the first visit of pregnant women with GDM in the present study were in the lower range compared to other studies (35, 48), which may mask the correlation. However, this study found that the EPR and B-FCC eating behavior were negatively associated with anxiety and depression, the B-FCC eating behavior was associated with lower BMI in the third trimester, the EPR eating behavior predicted lower anxiety and depression in the second trimester, and the RHSC eating behavior was associated with lower FPG in the third trimester. This complementary study examined the relationship between intuitive eating and anxiety and depression in pregnant women with GDM and the effect of the B-FCC subscale on pregnant women with GDM.

### 4.2 Potential mechanism

Although the effect of intuitive eating in GDM on weight and blood glucose during pregnancy remains to be investigated, intuitive eating is innate and cannot be ignored. The key feature of it is that it is based on a person’s physiological hunger and satiety signals, rather than external and emotional cues, and is essentially an “internal sensory” way of eating” (23). “Internal sensation” refers to the perception and processing of body signals based on afferent feedback from the brain, including the perception of the body’s physiological condition, as well as the representation of internal states in the context of ongoing activities (49–52). Anatomical studies have found that a class of afferent fibers monitors the physiological state of all internal organs of the body and converges on “inner sensory centers” in the insula cortex and generates conscious instinctive perceptions (51, 53). The visual state of the body is mapped to different brain regions, which are connected to a network of endoreceptor centers involved in representation and re-representation, as well as the integration of endoreceptor body signals with higher-order cognitive and emotional processes (50, 51, 54). The gastrointestinal system has demonstrated substantial (54–56), fairly stable, trait-like individual differences in the ability to process and perceive one’s body signals (i.e., endoreceptor sensitivity), and such differences have been shown to be reflected in differences in central endoreceptor network activity (52, 57). There is strong evidence that endosensory sensitivity is associated with emotional awareness of personal feelings (58, 59), emotional processing (60), and finer-grained behavioral self-regulation (61). Research has shown that an endosensory approach to eating can help people eliminate food guilt, listen and respond to moderate hunger and fullness signals in a confident, relaxed and enjoyable way, and enjoy the pleasure and satisfaction of food; and that people can consume more precise amounts of food based on hunger and fullness signals, resulting in a healthy eating style that is conducive to physical and mental health (24).

### 4.3 Strengths and limitations

The strength of this study was based on a clinically realistic longitudinal follow-up. This was the first study to investigate the association between intuitive eating and anxiety and depression in Chinese pregnant women with GDM. We measured pregnancy weight, blood glucose and other data before the women with GDM received dietary advice at their first visit to minimize confounding. In addition, we used a well-established and validated tool to measure intuitive eating during pregnancy. However, there were the following limitations of this study: (1) Pregnant women with GDM can know the GDM diagnosis in advance from the account tied to their mobile phones, so there will be a blank period from the diagnosis of GDM to the first visit, which may affect the level of anxiety and depression; (2) If possible, we obtained pre-pregnancy weight from the patient’s medical records, otherwise we relied on self-reports of pre-pregnancy weight, which may be limited. (3) The IES-2 total score was lacking in this study, and we did not include the UPE subscale due to the potential response bias described above. (4) The relatively small sample size limits our ability to generalize our findings. Therefore, Therefore, it was suggested that future studies could follow up the entire pregnancy from pre-pregnancy and use intuitive eating intervention to clarify the causal relationship between intuitive eating and anxiety, depression, pregnancy weight and blood glucose.

## 5 Conclusion

In this prospective cohort of women with GDM, the EPR and/or B-FCC and/or RHSC eating behavior were inversely associated with anxiety and depression, body weight, and blood glucose. Longitudinal associations indicated that higher scores on the EPR and/or RHSC subscales in the first GDM visit predicted lower third-trimester anxiety, depression and fasting glucose. These results suggest that practicing intuitive eating may be beneficial and that higher intuitive eating adherence can lead to lower levels of anxiety and depression and more ideal gestational weight and blood glucose values.

## Data availability statement

The original contributions presented in the study are included in the article/supplementary material, further inquiries can be directed to the corresponding author.

## Ethics statement

The studies involving humans were approved by Medical Ethics Committee of Fujian Maternal and Child Health Hospital (approval number: 2020Y9133). The studies were conducted in accordance with the local legislation and institutional requirements. The participants provided their written informed consent to participate in this study. Written informed consent was obtained from the individual(s) for the publication of any potentially identifiable images or data included in this article.

## Author contributions

XG: Data curation, Investigation, Methodology, Supervision, Writing – original draft, Writing – review & editing. QZ: Writing – review & editing. XC: Writing – review & editing. XJ: Funding acquisition, Resources, Writing – review & editing. YL: Data curation, Investigation, Writing – review & editing. YP: Writing – review & editing. JZ: Investigation, Writing – review & editing. GL: Investigation, Writing – review & editing.
